# The role of ACAID and CD4^+^CD25^+^FOXP3^+^ regulatory T cells on CTL function against MHC alloantigens

**Published:** 2008-12-19

**Authors:** Daniel R. Saban, Janet Cornelius, Sharmila Masli, Johannes Schwartzkopff, Maire Doyle, Sunil K. Chauhan, Ammon B. Peck, Maria B. Grant

**Affiliations:** 1 Department of Pathology, Immunology and Laboratory Medicine, University of Florida, Gainesville, FL; 2 Department of Pharmacology and Therapeutics, University of Florida, Gainesville, FL; 3 Schepens Eye Research Institute, Department of Ophthalmology, Harvard Medical School, Boston, MA; 4 Eye Hospital, Albert-Ludwigs University, Freiburg, Germany

## Abstract

**Purpose:**

Anterior chamber associated immune deviation (ACAID) is an antigen-specific form of peripheral immune tolerance that is induced to exogenous antigens placed in the ocular anterior chamber, which leads to a suppression in delayed-type hypersensitivity (DTH). Considerable work has been done on ACAID induction to major histocompatibility (MHC) alloantigens. However, its role on cytotoxic T lymphocyte (CTL) activity is currently unknown.

**Methods:**

C57BL/6 (H-2^b^) mice received an intracameral (IC) inoculation with BALB/c (H-2^d^) splenocytes. Splenic CD4^+^ and CD8^+^ T cell populations were characterized by flow cytometry and proliferation assays during induction and expression phases of ACAID. Percentages of CD4^+^CD25^+^FoxP3^+^ T regulatory cells (Treg) were also followed. Lastly, CTL function was measured at various time points during ACAID expression, and Treg were added to identify potential alterations in CTL function.

**Results:**

CD4^+^ and CD8^+^ T cell percentages and proliferation increased in the spleen during ACAID induction but then sharply decreased in response to an allospecific immunization. Expression of ACAID also exhibited a significant drop in CTL function. However, while Treg expansion was observed, these cells did not directly mediate the CTL inhibition.

**Conclusions:**

ACAID mediates an inhibition of CTL function against MHC alloantigens. Furthermore, we found that ACAID induction leads to the expansion and proliferation of CD4^+^ and CD8^+^ T cells while ACAID expression is associated with a diminishment in T cell percentages due to proliferation impairment. Lastly, Treg also expand during ACAID induction. However, our data suggest that Treg do not directly inhibit CTL activity.

## Introduction

Anterior chamber associated immune deviation (ACAID) is an antigen-specific form of peripheral immune tolerance that is induced against exogenous antigens placed in the ocular anterior chamber. Antigens inoculated into this immune privileged space are processed regionally by F4/80^+^ antigen presenting cells (APC) [1] that favor induction of tolerance through upregulated TGF-β production with a concomitant downregulation of CD40/CD40 ligand and interleukin (IL)-12 expression [2,3]. This APC population has been shown to migrate directly to the spleen [4] via vascularized structures within the iridocorneal angle (i.e., trabecular meshwork) [5] and with the aid of other accessory immune cells in the spleen, induces an antigen-specific state of immune tolerance. This form of tolerance is mainly characterized by a suppression of delayed-type hypersensitivity (DTH) reactions to the inoculated antigen [6].

Also relevant in ACAID is the activity of cytotoxic T lymphocytes (CTLs). These lymphocytes are CD8^+^ and are known to be important in killing microbially infected host cells and the destruction of tumor cells. The influence of ACAID expression on CTL function, however, is complex, and our current understanding remains incomplete. While it is known that intracameral inoculation with antigen-bearing tumors in mice induces ACAID, Streilein and coworkers [6,7] showed that CTL function in vitro nonetheless remains intact. Interestingly, however, Xu and Kapp [8] and Mckenna et al. [9] demonstrated that CTL function can be significantly impaired following the induction of ACAID when soluble antigens are inoculated intracamerally. Thus, one might conclude that the nature of the antigen used to induce ACAID impacts the manner by which it is processed and the subsequent effect on CTL.

CTL also plays a critical role in the immune rejection of allogeneic transplants, albeit not against major histocompatibility (MHC) alloantigens in corneal transplantation [10-12]. Indeed, corneal allografts induce ACAID to MHC alloantigens borne by the corneal allograft [13], which partially explains how these allografts enjoy higher survival rates relative to other forms of transplantation. However, whether ACAID is responsible for downregulating CTL function to MHC alloantigens is currently unknown, and this may be potentially important in further understanding increased corneal allograft survival. Moreover, further insight into ACAID mechanisms that may inhibit CTL against MHC alloantigens could potentially be an area applicable in suppressing CTL in other forms of transplantation as well.

We have previously reported that intracameral delivery of MHC allogeneic splenocytes (BALB/c) into C57BL/6 hosts induces ACAID [5]. In the present study, we have used this model to directly examine the role of ACAID on CTL function against allogeneic MHC targets. Furthermore, we specifically evaluated the possible relevance of CD4^+^CD25^+^FoxP3^+^ regulatory T cells (Treg) during the induction of ACAID and whether this population might be responsible for downregulating CTL function.

## Methods

### Animals and anesthesia

Female C57BL/6 and BALB/c mice six to eight weeks of age were purchased from Jackson Laboratories (Bar Harbor, Maine) and maintained under 12 h light/12 h dark cycles. Mice were provided water and food ad libitum. All procedures were performed under anesthesia, which included an intraperitoneal injection of ketamine (120 mg/kg whole bodyweight) and xylazine (20 mg/kg whole bodyweight) suspended in 100 μl of sterile Hank’s Balanced Salt Solution (HBSS). Mice were euthanized by cervical dislocation following anesthetization. Throughout these studies, mice were handled according to guidelines established by the ARVO Statement for the Use of Animals in Ophthalmic and Vision Research and Public Health Policy on Humane Care and Use of Laboratory Animals (US Public Health Review). These studies were approved by the University of Florida’s IACUC.

### Intracameral inoculation and subcutaneous immunizations

The procedures for intracameral (IC) inoculation have been described in detail elsewhere [5]. In brief, the cornea was punctured with a 30 gauge insulin syringe (BD and Co., Franklin Lakes, NJ) and approximately 2 μl of air was dispensed into the anterior chamber. An allogeneic splenocyte suspension containing 1×10^6^ cells per 2 µl in sterile HBSS was then delivered into the anterior chamber. Microinjections were administered with a pulled 10 μl glass micropipette (Drummond Scientific Co., Broomall, PA) fitted into a sterile infant feeding tube (No. 5 French; Cutter Laboratories, Inc., Berkeley, CA), which was attached to a 1 ml insulin syringe. Immunizations consisted of allogeneic splenocytes in sterile HBSS. Restraining the mice, immunizations were delivered subcutaneously to the base of the tail using a sterile 30 gauge, 25 μl Hamilton syringe (Hamilton Co. Inc., Whittier, CA) carefully avoiding the tail vein.

### Delayed-type hypersensitivity assay

This assay has been previously described elsewhere [5]. In brief, γ-irradiated (3000 R) splenocytes (10^6^ cells per 10 µl) were loaded into a 30 gauge, 1 ml insulin syringe, which was inserted intradermally into the host ear pinnae. The needle was then advanced toward the outer edge of the pinnae, and cell suspensions were slowly dispensed. Specific ear swelling was measured with a micrometer (Mitutoyo, Aurora, IL) at the location where the bolus was dispensed. These measurements were administered before the challenge as a baseline, 24 h post ear challenge, and 48 h post ear challenge. At each of these time points, three individual measurements were taken per ear and averaged. Peak DTH responses (either 24 h or 48 h) were used to calculate specific ear swelling, which equals mean peak measurements subtracted by the mean baseline.

### Flow cytometry

Characterization of T cell populations was performed on splenocytes from freshly sacrificed mice or on cultured splenocytes. Cells were thoroughly washed in 0.5% BSA and incubated with FC-receptor antibody (CD16/CD32) in the dark at 4 °C for 15 min as per manufacturer’s instruction (Becton Dickinson PharMingen). Cells were then double-stained with FITC-conjugated anti-CD4 or PE-conjugated anti-CD8 antibodies for 30 min in the dark at 4 °C according to the manufacturer’s instruction (BD PharMingen). Aliquots were also made for appropriate isotype controls. For intracellular staining, thoroughly washed cells first underwent FC-receptor blockade and cell surface staining with PE-conjugated anti-CD4 and PECy7-conjugated anti-CD25 antibodies (eBioscience, San Diego, CA) for 30 min in the dark at 4 °C. Fixation and permeabilization was performed overnight (eBioscience) followed by intracellular staining for 30 min with anti-Foxp3 antibody at 4 °C.

### T cell proliferation

A small aliquot was taken from freshly harvested experimental host (C57BL/6) splenocytes to enumerate CD4^+^ and CD8^+^ T cell percentages before allostimulation. The remainder was then stimulated in bulk using BALB/c allosplenocytes (γ-irradiated at 3,000 R) at a 1:1 ratio to measure T cell proliferation. An upright T 25 flask with 10 ml of Click’s medium (EHAA; Sigma- Aldrich, St. Louis, MO) supplemented with10% fetal bovine serum was used to culture 50.0×10^6^ host splenocytes co-incubated with an equal number of BALB/c stimulator cells at 37 °C for five days. Flow cytometry was subsequently used to enumerate the percentages of emanating CD4^+^ and CD8^+^ T cells. Ratios of the respective T cell percentage after allostimulation over the respective percentage enumerated before allostimulation yielded the proliferation index.

### Fluorolysis assay for CTL function and relevance of Treg

The fluorolysis assay for CTL function in vitro has been previously described [14]. Briefly, harvested splenocytes from C57BL/6 were stimulated at a 1:1 ratio with BALB/c allosplenocytes (γ-irradiated) and maintained for five days in modified EHAA at 37 °C. Target cells were harvested from allogeneic, NOD/LtJ-eGFP^+^ (H-2K^d^) mice and stimulated with LPS (5 μg/ml) for five days at 37 °C. Responder and target cells were collected and enumerated via trypan blue exclusion. Responder cells were re-plated in triplicate using a 96 well plate and co-cultured with target cells at ratios of 10:1, 2.5:1, and 1:1 (responder to target) for 4 h at 37 °C. After incubation, harvested target cells were thoroughly washed and were enumerated via flow cytometry based on eGFP^+^ cells, which yielded the target cell survival, a value that is inversely proportional to CTL function.

We also assayed the relevance of Treg in CTL function. Naïve T cells were stimulated in vitro and re-plated at a ratio of 2.5 to 1 with eGFP^+^ target cells. Thus, 2.5×10^5^ responders with 1.0×10^5^ targets were plated in a 96 well plate for a 4 h CTL assay. Treg were magnetically sorted based on CD4^+^CD25^+^ markers from the spleens of ACAID-induced mice and 0.35×10^5^ were added, or cells not added as a control, to the CTL assay. After incubation, harvested target cells were thoroughly washed and were enumerated via flow cytometry based on eGFP^+^ cells to yield the target cell survival.

### Statistics

Data are presented as the standard error of the mean, and statistical analyses included Student’s *t*-test or ANOVA as indicated in respective figure captions. p values less than 0.05 were considered statistically significant.

## Results

### ACAID is detectable out to 24 days following intracameral inoculation

**Figure 1:**
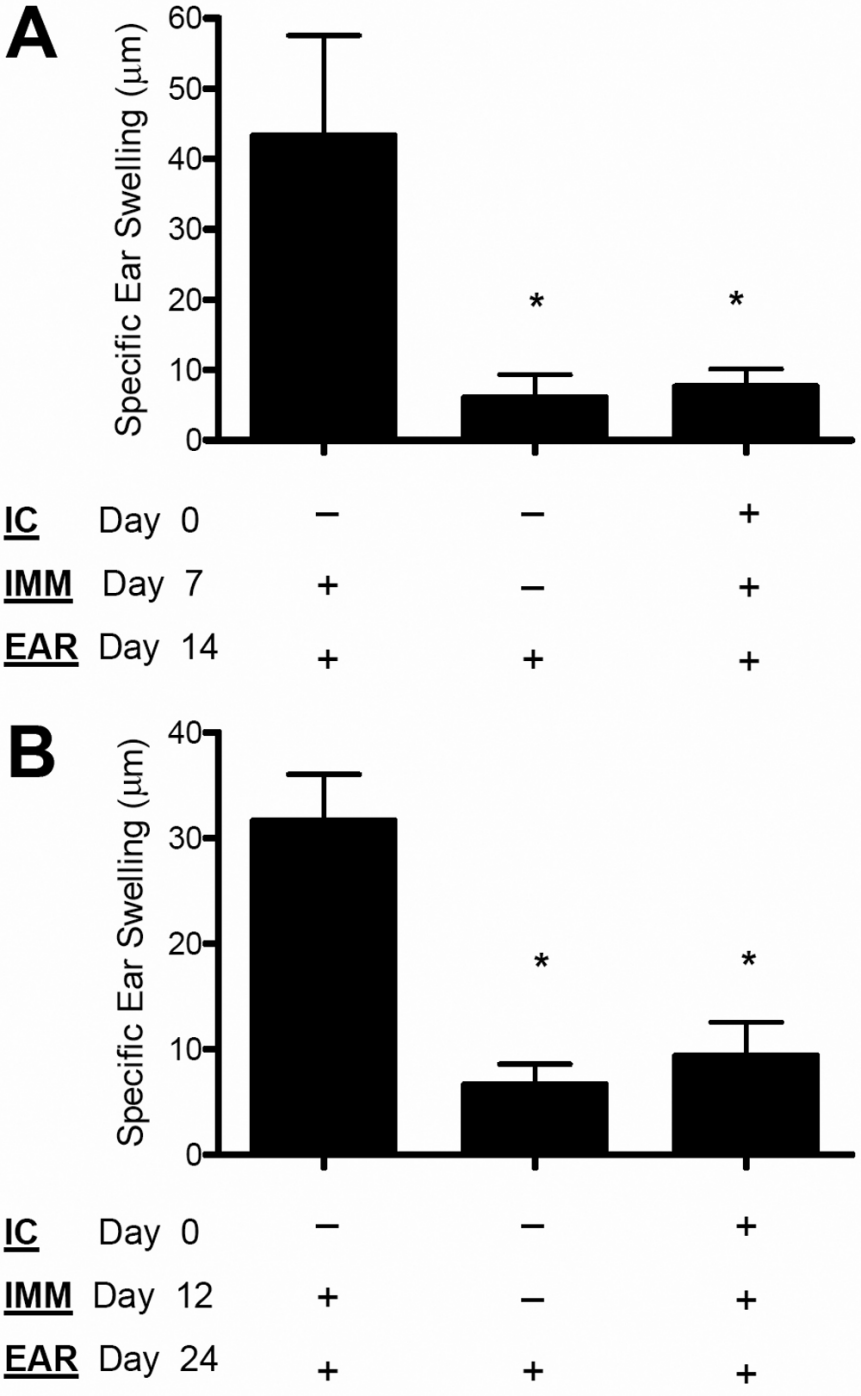
ACAID is detectable up to 24 days following intracameral inoculation. **A**: ACAID induction is typically tested approximately 14 days post intracameral inoculation. C57BL/6 mice were intracamerally (IC) inoculated with allogeneic BALB/c splenocytes to induce ACAID. A subsequent immunization (IMM) with BALB/c splenocytes was administered subcutaneously on day 7 to prime IC inoculated mice. Naïve mice were similarly primed as a positive control. Following the challenge with BALB/c splenocytes delivered subcutaneously to the host ear pinnae (EAR), specific ear swelling was then measured to assess DTH. Naïve mice were also challenged as a negative control. **B**: ACAID was also detected 24 days post IC inoculation. In other mice, IMM was administered on day 12, and DTH was subsequently tested on day 24. Each group consisted of at least five mice or more, and an asterisk indicates statistical significance (p<0.05) calculated via ANOVA.

In the current study, C57BL/6 hosts (*H-2^b^*) were inoculated IC with spleen cells freshly isolated from allogeneic BALB/c mice (*H-2^d^*), a recipient-donor combination previously shown to induce ACAID [5]. Following IC inoculation to induce ACAID, this response is classically detected by first immunizing the recipient mice seven days post IC inoculation with the inducing antigen. Subsequently, approximately seven days after immunization, inhibition of DTH can be observed to an antigen-specific challenge delivered subcutaneously to the recipient ear pinnae. For the present study, we elected to delay these intervals typically used to study ACAID to facilitate our study of T cell immunity. Thus, immunization was given 12 days after IC inoculation, and the antigenic challenge was subsequently delivered after another 12 days. As presented in Figure 1, this alteration in the protocol did not affect the ability of ACAID to suppress DTH responses as this form of tolerance was detectable up to 24 days post IC inoculation.

### Characterizing responses by T cell subpopulations during the induction and expression of ACAID

We first investigated whether CD8^+^ T cells are expanding during the induction and/or expression of ACAID to MHC alloantigens as this could potentially confer the activation of a CTL response in the current model. Moreover, since T helper cells are indeed important in the activation/differentiation of CTL, CD4^+^ T cells were also assessed. Flow cytometry was used to measure CD4^+^ and CD8^+^ T cell percentages in host spleens, the critical site for the activation of ACAID [4]. C57BL/6 mice were inoculated IC with BALB/c allosplenocytes, and their spleens were collected 4, 8, and 12 days post IC inoculation in addition to 16, 20, and 24 days post IC inoculation. The initial three time points (4, 8, and 12 days) allowed us to examine the induction of ACAID. The latter time points (days 16, 20, and 24), which followed the delivery of an immunization, allowed us to examine the expression of ACAID.

**Figure 2:**
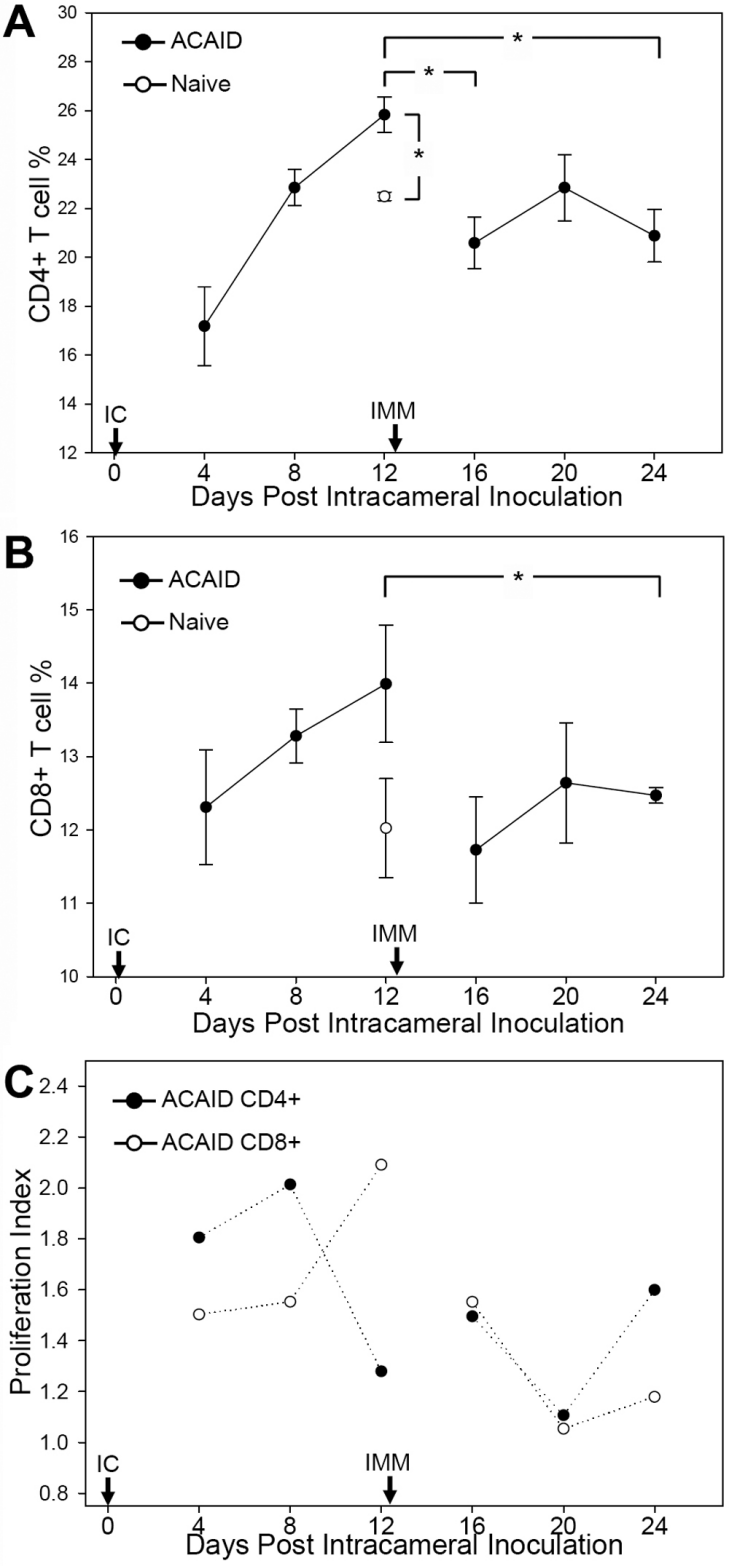
In vivo percentages of splenic CD4^+^ and CD8^+^ T cells and their proliferative responses in vitro during the induction and expression of ACAID. Induction of ACAID to MHC alloantigens is associated with the expansion of CD4^+^ (**A**) and CD8^+^ T (**B**) cells while expression of ACAID is associated with a reduction of T cell percentages. C57BL/6 mice were IC inoculated with allogeneic BALB/c splenocytes to examine the induction of ACAID and was followed by immunization (IMM) with BALB/c splenocytes to examine the expression of ACAID. Splenocytes were harvested at the indicated time points (n=3 per time point) for enumeration of T cell percentages via flow cytometry. Naïve spleens were similarly assessed and presented graphically on day 12. An asterisk indicates statistical significance (p<0.05) calculated via Student’s *t*-test. **C**: Induction of ACAID is associated with higher proliferation levels while expression of ACAID is associated with lower proliferation levels. T cells were harvested from hosts (n=3 per time point) at the indicated time points and stimulated in vitro to recall alloantigen (BALB/c).

We found that the general patterns of CD4^+^ T cell percentages (Figure 2A) are somewhat similar to those of CD8^+^ T cells (Figure 2B) during the induction and expression of ACAID. During the induction of ACAID, there is a substantial increase that takes place in both CD4^+^ (p<0.01) and CD8^+^ T cells peaking on day 12 when comparing to respective splenic T cell populations in naïve mice. Comparison of these respective peaks on day 12 to subsequent time points following immunization then indicates a decrease in CD4^+^ and CD8^+^ T cell populations. Statistically significant decreases during ACAID expression occur in CD4^+^ T cells on days 16 (p<0.05) and 24 (p<0.05; Figure 2A), and significant decreases in CD8^+^ T cells occur on day 24 (p=0.044; Figure 2B). Thus, despite the considerably delayed delivery of immunization on day 12 post IC inoculation, only following immunization does a diminishment in the splenic T cell percentage occur. This suggests that it is the immunization of IC inoculated mice that leads to the diminishment in splenic T cell percentage and potentially signifies an ACAID-mediated regulation of immune responses to the immunization.

We then assessed whether T cell proliferation plays a role in these observed alterations of CD4^+^ and CD8^+^ T cell percentages during the induction and expression of ACAID. Proliferative responses were therefore tested in vitro by using recall alloantigen (BALB/c) stimulation of T cells harvested at the indicated time points. T cells were left unfractionated to maintain their proportionality in situ, which allows us to recapitulate and evaluate their net effect on their proliferative responses. Using this analysis, we found that the general trend of proliferation for both T cell populations is somewhat reflective of their percentage alterations observed in the spleen (Figure 2C). The induction of ACAID is associated with peak CD4^+^ (on day 8) and CD8^+^ T cell proliferation (on day 12) while the expression of ACAID is associated with a downregulation of proliferative responses in these populations, particularly on day 20, thus potentially indicating ACAID-mediated regulation of immune responses to the immunization.

**Figure 3:**
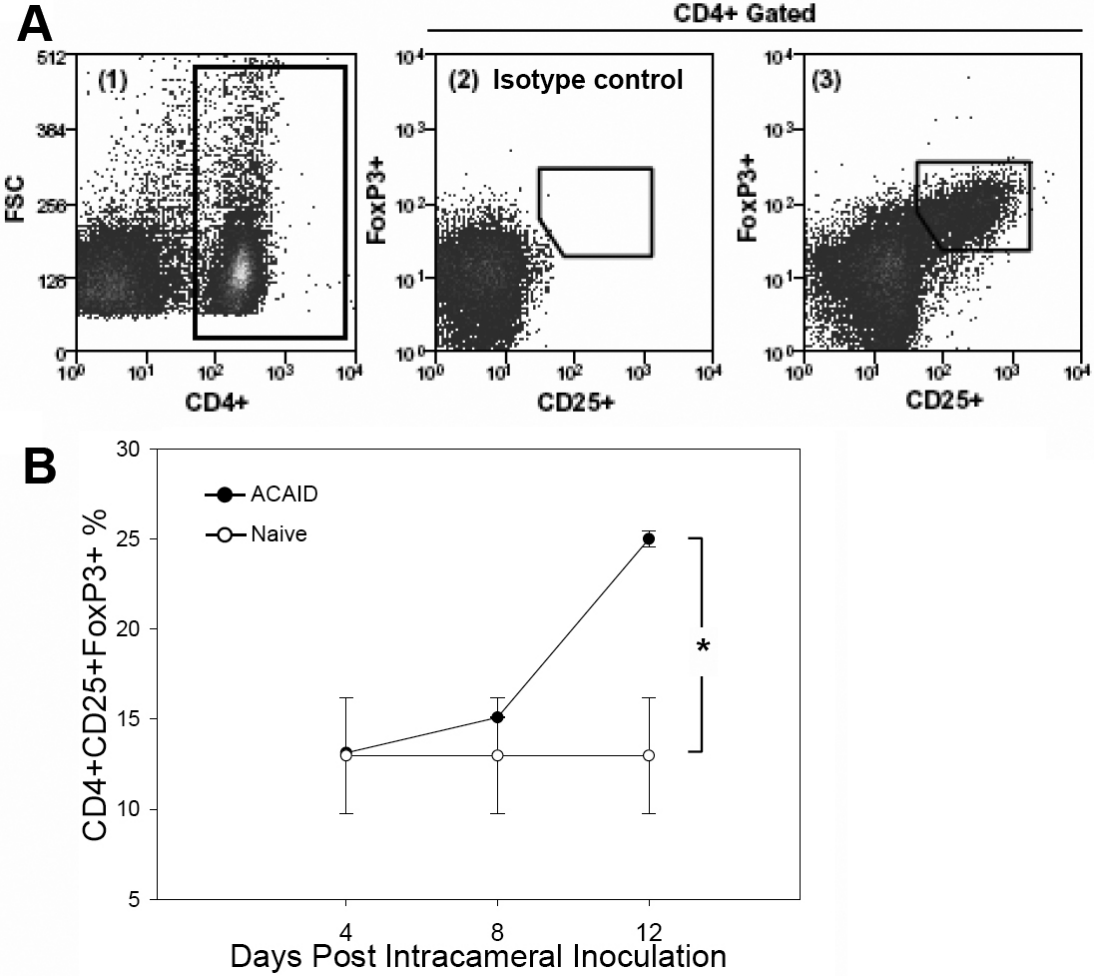
Intracameral inoculation leads to an increase in CD4^+^CD25^+^FoxP3^+^ Treg. **A**: Flow cytometry was used to enumerate Treg in the spleen. Triple-stained splenocytes for CD4^+^, CD25^+^, and FoxP3^+^ were first gated on CD4^+^ (left) and then measured for CD25 and FoxP3 (center, right). **B**: ACAID induction leads to an increase in Treg in the spleen. Spleens were harvested and enumerated for Treg percentages in naïve mice or in IC inoculated mice on days 4, 8, and 12 (n=3 per time point). An asterisk indicates a statistically significant increase (p<0.5) in percentage over the naïve control. The statistical significance was calculated via Student’s *t*-test.

The reduced levels of proliferation, especially in CD4^+^ T cells, suggested a role for CD4^+^CD25^+^FoxP3^+^ T regulatory cells (Treg) as these cells are well known for their ability to suppress T cell proliferation [15]. To examine this possibility, spleen cells from naïve mice or from IC inoculated mice on days 4, 8, and 12, were collected, and the CD4^+^CD25^+^FoxP3^+^ T cell populations enumerated using flow cytometry (Figure 3A). These time points allowed us to focus on the induction of ACAID, particularly where the marked suppression of CD4^+^ proliferation was observed around day 12. As presented in Figure 3B, IC inoculated mice exhibited significantly increased levels of Treg cells on day 12 (p<0.05) relative to those exhibited by naive mice.

### The effect of ACAID induction on CTL and the relevance of Treg function

The reduction of CD8^+^ T cell percentages in the spleen in addition to the suppression of their proliferation points to the possibility that important functional changes may be taking place in effector CD8^+^ T cells during the expression of ACAID. We were therefore interested in testing CTL function following the immunization of IC inoculated mice. This was tested by the fluorolysis assay [14] using recall in vitro allostimulated (H-2^d^) T cells co-cultured with GFP^+^ allospecific (H-2K^d^) target cells at various responder:target cell ratios. Flow cytometry was subsequently used to enumerate target cell survival, an assay in which values are inversely proportional to CTL function. Baseline CTL activity was established with in vitro stimulated T cells harvested from naïve mice while the positive control employed in vitro stimulated T cells from immunized mice harvested 4, 8, and 12 days post immunization. T cells were similarly harvested from IC inoculated mice 4, 8, and 12 days post immunization.

**Figure 4:**
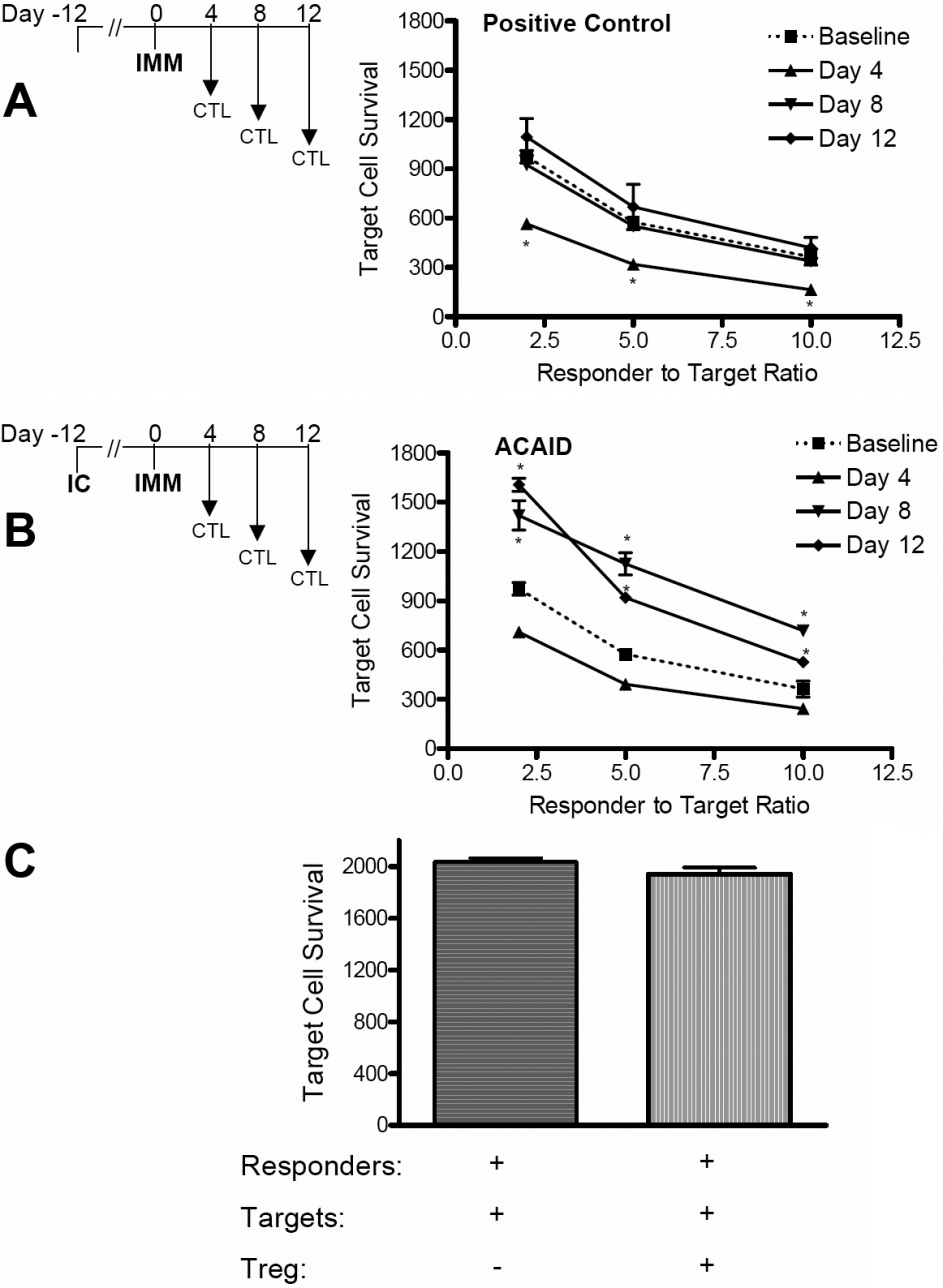
ACAID suppresses CTL function, and this suppression does not appear to be directly mediated by CD4^+^CD25^+^FoxP3^+^ Treg. Splenocytes were harvested on days 4, 8, and 12 (n=3 per time point) post immunization from positive control mice (**A**) or ACAID expressing mice (**B**). Following their alloantigen stimulation in vitro, responder cells were plated with allospecific eGFP^+^ target cells at ratios of 2.5, 5, and 10 to 1 (responder to target) in triplicate wells. Baseline activity was obtained with in vitro allostimulated naïve splenocytes. Flow cytometry was used to enumerate target cell survival, values that are inversely proportional to CTL function. An asterisk indicates statistical significance as calculated by ANOVA. **C**: ACAID-induced Treg do not directly suppress CTL function. Baseline CTL cultures were incubated at a 2.5:1 ratio and splenic Treg magnetically sorted from ACAID-induced mice were added to CTL assays. No statistical (NS) difference was observed as calculated via Student’s *t*-test.

As presented in Figure 4A, target cell survival in the positive controls was significantly lower than baseline on day 4 post immunization, indicative of increased CTL activity (p<0.01), while target cell survival at days 8 and 12 proved statistically similar to baseline. Interestingly, following the immunization of IC inoculated mice, a marked increase in target cell survival (p<0.01) is exhibited on days 8 and 12 (Figure 4B) despite the fact that T cells harvested from day 4 post immunization exhibited activities lower than baseline. These data show that ACAID suppresses the CTL response against allo-MHC target cells. To then test whether ACAID-induced Treg cells are directly involved in this suppression of CTL activity, Treg from ACAID-induced mice were added to the CTL cultures. As shown in Figure 4C, no significant effect on target cell survival was observed with the addition of ACAID-induced Treg cells, suggesting that these cells do not directly suppress the CTL activity in ACAID.

## Discussion

This is the first study to show that ACAID mediates an inhibition of CTL function against allogeneic MHC target cells, as splenic CD8^+^ T cells exhibited impaired responses to subsequent immunization with alloantigens. Furthermore, we found that while ACAID induction leads to the expansion and proliferation of CD4^+^ and CD8^+^ T cells, ACAID expression is associated with a reduction in T cell numbers and proliferation potential toward the immunizing alloantigens. Lastly, we observed an expansion of CD4^+^CD25^+^FoxP3^+^ Treg during the induction of ACAID. However, our data suggest that this Treg population probably does not directly inhibit CTL activity.

In support of our findings, previous studies have demonstrated that ACAID induced with soluble antigens can lead to an inhibition of CTL function in vitro [8,9]. In contrast, it has been shown that ACAID induced with exogenous tumors may not inhibit in vitro CTL function [6,7,16,17]. These disparate responses in CTL activity associated with ACAID are postulated to be a result of the nature of the antigen (i.e., soluble versus cellular antigen) employed to induce ACAID. Antigen-bearing tumor cells are capable of migrating to the spleen following IC delivery [6], thereby circumventing the processing by the ocular APC considered essential for the induction of ACAID and directly priming the CTL response. In contrast, soluble alloantigens inoculated IC would be processed by ocular APC, thereby skewing the response to ACAID and subsequent tolerance.

In the current study, however, we found that allogeneic spleen cells, which include allo-APC capable of directly presenting antigen via class I, induces a form of ACAID that leads to reduced CTL function. Moreover, we have previously shown that following IC inoculation, allogeneic spleen cells can indeed exit the eye intact via the iridocorneal angle [5], providing the opportunity for direct presentation by inoculated allogeneic APC to the host. Thus, our current data refute the notion that CTL inhibition in ACAID is exclusively reserved for soluble antigens and further suggests that direct presentation by IC inoculated cells can lead to CTL impairment associated with ACAID induction. Whether this event may be restricted to APC that become toleragenic with exposure to high levels of TGF-β in the ocular anterior chamber [16] requires further investigation.

We also observed that while CD4^+^ and CD8^+^ T cells undergo expansion in the spleen during the induction of ACAID, their detectable numbers are sharply reduced in response to a subsequent immunization. Because this observation is consistent with decreased proliferation levels in both CD4^+^ and CD8^+^ T cell populations, it seemed logical that this decline was due to impairment in T cell proliferation rather than simply the egress of T cells from the spleen. Not surprisingly, therefore, that we observed a concomitant expansion of CD4^+^CD25^+^FoxP3^+^ Treg cells during ACAID induction, pointing to the possibility that these cells play an important role in suppressing T cell proliferation in the spleen, this is in line with both Keino et al. [18] and Zhang et al. [19].  Furthermore, it is likely that these are inducible Treg rather than natural Treg in mediating ACAID, as previously described by Keino et al.  [18].

However, our results suggest that while direct regulation of CTL function in vitro by other T cells (i.e., δγ T cells) has been reported [8], CD4^+^CD25^+^FoxP3^+^ Treg cells induced by ACAID do not directly suppress CTL function. It is more likely that these ACAID-induced Treg cells suppress CTL by an indirect means, e.g., by inhibiting helper CD4^+^ T cell function, which is important in promoting CD8^+^ T cell differentiation and effector activity. Although this latter hypothesis is attractive, it will be necessary to prove such cellular interactions exist in ACAID-induced suppression of CTL activity. It is also important to point out that CD8^+^ Treg, known to suppress the expression of DTH in ACAID [20], were also presumably generated in the current study. Future work will be required to define their potential role, if any, in the suppression of CTL responses observed in this study.

In summary, we report that IC induction of ACAID is detectable up to at least 24 days, and this is associated with the suppression of CTL function to MHC allogeneic targets. Furthermore, these data suggest that suppression of CTL activity is not directly mediated by CD4^+^CD25^+^FoxP3^+^ Treg generated during the induction of ACAID. Understanding this aspect is important as it raises questions as to whether these data possibly explain why CTL activity is minimal in corneal allograft rejection [10-12] as these grafts indeed lead to the induction of ACAID [13]. Lastly, the current model may offer further insight into ACAID mechanisms that inhibit CTL against MHC alloantigens, an area that is potentially applicable in suppressing CTL in other forms of transplantation as well.

## References

[1] Wilbanks GA, Streilein JW (1991). Studies on the induction of anterior chamber-associated immune deviation (ACAID). 1. Evidence that an antigen-specific, ACAID-inducing, cell-associated signal exists in the peripheral blood.. J Immunol.

[2] Cousins SW, McCabe MM, Danielpour D, Streilein JW (1991). Identification of transforming growth factor-beta as an immunosuppressive factor in aqueous humor.. Invest Ophthalmol Vis Sci.

[3] Takeuchi M, Alard P, Streilein JW (1998). TGF-beta promotes immune deviation by altering accessory signals of antigen-presenting cells.. J Immunol.

[4] Streilein JW, Niederkorn JY (1981). Induction of anterior chamber-associated immune deviation requires an intact, functional spleen.. J Exp Med.

[5] Saban DR, Elder IA, Nguyen CQ, Smith WC, Timmers AM, Grant MB, Peck AB (2008). Characterization of intraocular immunopathology following intracameral inoculation with alloantigen.. Mol Vis.

[6] Niederkorn JY, Streilein JW (1983). Alloantigens placed into the anterior chamber of the eye induce specific suppression of delayed-type hypersensitivity but normal cytotoxic T lymphocyte and helper T lymphocyte responses.. J Immunol.

[7] Ksander BR, Streilein JW (1989). Analysis of cytotoxic T cell responses to intracameral allogeneic tumors.. Invest Ophthalmol Vis Sci.

[8] Xu Y, Kapp JA (2002). Gammadelta T cells in anterior chamber-induced tolerance in CD8(+) CTL responses.. Invest Ophthalmol Vis Sci.

[9] McKenna KC, Xu Y, Kapp JA (2002). Injection of soluble antigen into the anterior chamber of the eye induces expansion and functional unresponsiveness of antigen-specific CD8+ T cells.. J Immunol.

[10] Sano Y, Ksander BR, Streilein JW (2000). Analysis of primed donor-specific T cells in recipient mice bearing orthotopic corneal allografts.. Transplantation.

[11] Sano Y, Streilein JW, Ksander BR (1999). Detection of minor alloantigen-specific cytotoxic T cells after rejection of murine orthotopic corneal allografts: evidence that graft antigens are recognized exclusively via the “indirect pathway”.. Transplantation.

[12] Hegde S, Niederkorn JY (2000). The role of cytotoxic T lymphocytes in corneal allograft rejection.. Invest Ophthalmol Vis Sci.

[13] Sonoda A, Sonoda Y, Muramatu R, Streilein JW, Usui M (2000). ACAID induced by allogeneic corneal tissue promotes subsequent survival of orthotopic corneal grafts.. Invest Ophthalmol Vis Sci.

[14] Kienzle N, Olver S, Buttigieg K, Kelso A (2002). The fluorolysis assay, a highly sensitive method for measuring the cytolytic activity of T cells at very low numbers.. J Immunol Methods.

[15] Hori S, Nomura T, Sakaguchi S (2003). Control of regulatory T cell development by the transcription factor Foxp3.. Science.

[16] Hara Y, Okamoto S, Rouse B, Streilein JW (1993). Evidence that peritoneal exudate cells cultured with eye-derived fluids are the proximate antigen-presenting cells in immune deviation of the ocular type.. J Immunol.

[17] McKenna KC, Kapp JA (2004). Ocular immune privilege and CTL tolerance.. Immunol Res.

[18] Keino H, Takeuchi M, Kezuka T, Hattori T, Usui M, Taguchi O, Streilein JW, Stein-Streilein J (2006). Induction of eye-derived tolerance does not depend on naturally occurring CD4+CD25+ T regulatory cells.. Invest Ophthalmol Vis Sci.

[19] Zhang H, Yang P, Zhou H, Meng Q, Huang X. (2008). Involvement of Foxp3-expressing CD4+ CD25+ regulatory T cells in the development of tolerance induced by transforming growth factor-beta2-treated antigen-presenting cells..

[20] Wilbanks GA, Streilein JW (1990). Characterization of suppressor cells in anterior chamber-associated immune deviation (ACAID) induced by soluble antigen. Evidence of two functionally and phenotypically distinct T-suppressor cell populations.. Immunology.

